# Case Report: Balanced Reciprocal Translocation t (17; 22) (p11.2; q11.2) and 10q23.31 Microduplication in an Infertile Male Patient Suffering From Teratozoospermia

**DOI:** 10.3389/fgene.2022.797813

**Published:** 2022-05-26

**Authors:** Shan Huang, Huiling Wu, Yunwei Qi, Liqiang Wei, Xiaodan Lv, Yu He

**Affiliations:** Department of Clinical Laboratory, The First Affiliated Hospital of Guangxi Medical University, Nanning, China

**Keywords:** male infertility, balanced reciprocal translocation, microduplication, copy number variations, teratozoospermia

## Abstract

Two chromosomal abnormalities are described in an infertile man suffering from teratozoospermia: balanced reciprocal translocation t (17; 22) (p11.2; q11.2) and a microduplication in the region 10q23.31. Twenty genes located on the breakpoints of translocation (e.g., *ALKBH5*, *TOP3A*, *SPECC1L*, and *CDC45*) are selected due to their high expression in testicular tissues and might be influenced by chromosome translocation. Four genes located on the breakpoints of microduplication including *FLJ37201*, *KIF20B*, *LINC00865*, and *PANK1* result in an increased dosage of genes, representing an imbalance in the genome. These genes have been reported to be associated with developmental disorders/retardation and might be risk factors affecting spermatogenesis. Bioinformatics analysis is carried out on these key genes, intending to find the pathogenic process of reproduction in the context of the translocation and microduplication encountered in the male patient. The combination of the two chromosomal abnormalities carries additional risks for gametogenesis and genomic instability and is apparently harmful to male fertility. Overall, our findings could contribute to the knowledge of male infertility caused by genetic factors.

## Introduction

Fertility rates have been declining worldwide over the past decade. Male infertility has been garnering increasing attention (Agarwal et al., 2021). Several health conditions and lifestyle factors (e.g., obesity, smoking, and alcoholism) may affect fertility. However, around 40% of infertile men are underdiagnosed, which might be attributed to underlying genetic causes (Krausz and Riera-Escamilla, 2018).

Structural abnormalities of chromosomes in sperm increase the risk of aneuploidy and unbalanced chromosomal complements in the fetus (Krausz and Riera-Escamilla, 2018) (Hofherr et al., 2011). The reciprocal translocation t(17; 22) has been documented in two infertile men, but with different chromosomal breakpoints (Geneix et al., 2002) (Ji Won Kim et al., 2011). In one of those two patients, a meiotic segregation pattern of 3:1 was reported, which is unusual. Reports of the long arm of chromosome 10 microduplication combined with another chromosomal abnormality are uncommon. Chromosomal changes (particularly those involving segmental duplications) can contribute to high genomic instability (Hofherr et al., 2011) (Manvelyan et al., 2007). Furthermore, sperm morphology is a prominent component of semen analyses and is important for the care of infertile couples and their choice of assisted reproductive technology (ART).

We studied a man with teratozoospermia with regard to cytogenetics and copy number variations (CNVs) whose wife suffered a miscarriage. A balanced reciprocal translocation of t (17; 22) (p11.2; q11.2) and a microduplication in the region 10q23.31 were found. With respect to ART, it is crucial to obtain information about the genetic causes of male infertility because these defects can be transmitted across generations.

## Materials and Methods

### Ethical Approval of the Study Protocol

The study protocol was approved by the Ethics Committee of the First Affiliated Hospital of Guangxi Medical University (Nanning, China). Written informed consent was obtained from the patient.

### Semen Analyses

In brief, semen analyses were undertaken following the method recommended in the fifth edition of the WHO laboratory manual for the examination and processing of human semen (Organization, 2010). Semen samples were collected twice at an interval of 7 days after abstinence from coitus. Sperm motility was calculated by counting ≥200 sperms.

### Karyotype Analyses

Peripheral blood of the patient was treated with colchicine for 1.5 h, incubated in a hypotonic solution, and fixed with methanol–acetic acid solution. Karyotype analyses were carried out with Giemsa banding of chromosomes in metaphase (400–550 bands). Twenty metaphase counts and eight karyotypes were analyzed. Karyotypes were described in accordance with the International Human Cytogenetic Nomenclature System (2020) (Jean McGowan-Jordan and Moore, 2020).

### Molecular Analyses

Studies on microdeletion of the Y-chromosome were undertaken using a multiplex polymerase chain reaction (PCR) amplification-based method (Simoni et al., 1999). In brief, DNA was extracted using standard methods, followed by two separate multiplexed PCRs. The patient was considered to have Y-chromosome microdeletion if a deletion was detected in at least one marker within the verification set.

### Next Generation Sequencing

CNVs were analyzed on the NovaSeq 6000 platform (Illumina, San Diego, CA, United States). Experiments were carried out with the NovaSeq 6000 S4 Reagent Kit and the S4 flow cell in accordance with manufacturer protocols. The PE150 system within the NovaSeq 6000 kit was employed for genotype calling, quality control, and CNV identification. For the annotation of genes in deleted or duplicated genomic segments, the public databases Database of Genomic Variants (DGV; www.dgv.tcag.ca/dgv/app/home), Clinical Genome Resource (ClinGen; www.clinicalgenome.org/data-sharing/clinvar/), Online Mendelian Inheritance in Man (OMIM; www.omim.org/), Genome Aggregation Database (gnomAD; www.gnomad.broadinstitute.org/), GeneReviews (www.ncbi.nlm.nih.gov/books/NBK1116/), and Database of Chromosomal Imbalance and Phenotype in Humans Using Ensembl Resources (DECIPHER; www.deciphergenomics.org/) were used.

### Bioinformatics Analysis

Data on the expression of candidate genes in normal tissues were extracted from the National Center for Biotechnology Information (www.ncbi.nlm.nih.gov/gene/) and Human Protein Atlas (www.proteinatlas.org) (Uhlen et al., 2015). The functions of these genes were obtained from the literature and neXtProt knowledge base (www.nextprot.org) (Zahn-Zabal et al., 2020). The interaction network of genes was determined using GeneMANIA (www.genemania.org/) (Franz et al., 2018). Positional plots of the microduplications on 10q23.31 regions were obtained from the Genome Browser (www.genome.ucsc.edu).

## Results

A 35-year-old man arrived at the Department of Prenatal and Genetic Diseases within the First Affiliated Hospital of Guangxi Medical University for consultation regarding a miscarriage suffered by his wife. A questionnaire was used to record information on height, bodyweight, occupation, lifestyle, family history, exposure to radiation/toxins, and trauma. His wife got pregnant naturally and exhibited a well-developed female phenotype. Results of routine examinations were taken from electronic medical records.

**FIGURE 1:**
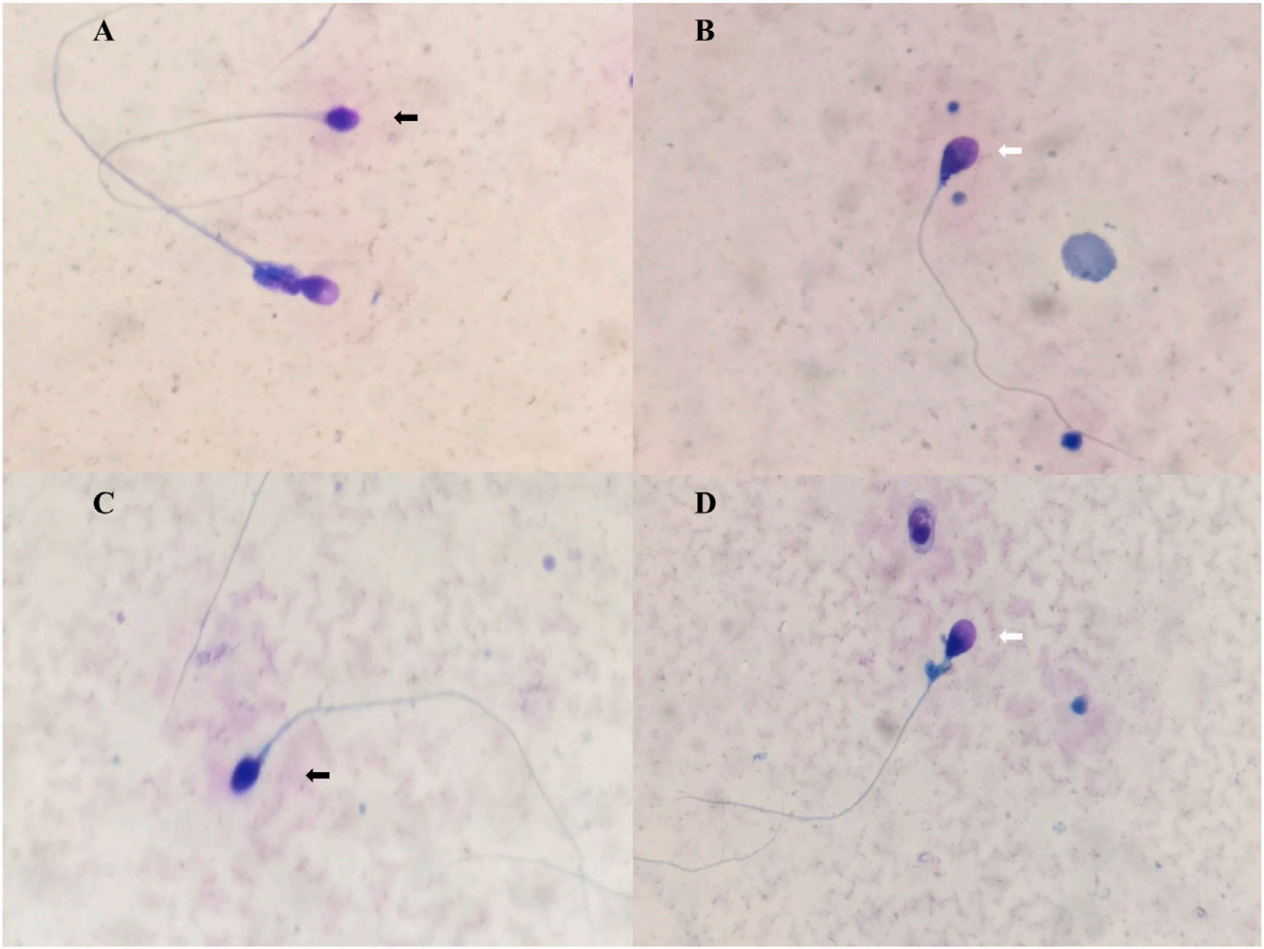
Morphology sperm images of the case. **(A,C)** The black arrow indicates a rounded acrosomeless head. The white arrow indicates that the pyriform sperm head and midpiece comments are thick. **(B,D)** The black arrow indicates round without acrosome. The white arrow indicates a pyriform sperm head, and less than 40% of the head is occupied by the acrosome.

For the patient, levels of follicle-stimulating hormone, luteinizing hormone, estradiol, prolactin, and testosterone in serum were found to be within the normal range. The testicular volume was normal, and other disorders affecting the reproductive system were not observed. Semen analyses of two samples revealed asthenozoospermia [normal sperm count (≥15×10^6^/ml) but a lower percentage of sperm with progressive motility (<32%)] and teratozoospermia (abnormal sperm morphology >96%) (Supplementary Table S1). Conspicuous head-shaped and acrosomal anomalies of sperm were observed (Figure 1). Many sperm heads were absent or minute acrosome, whose acrosome/sperm head ratio was less than 40%. Some sperms were pyriform or irregularly ovoid and had small acrosomes.

**FIGURE 2:**
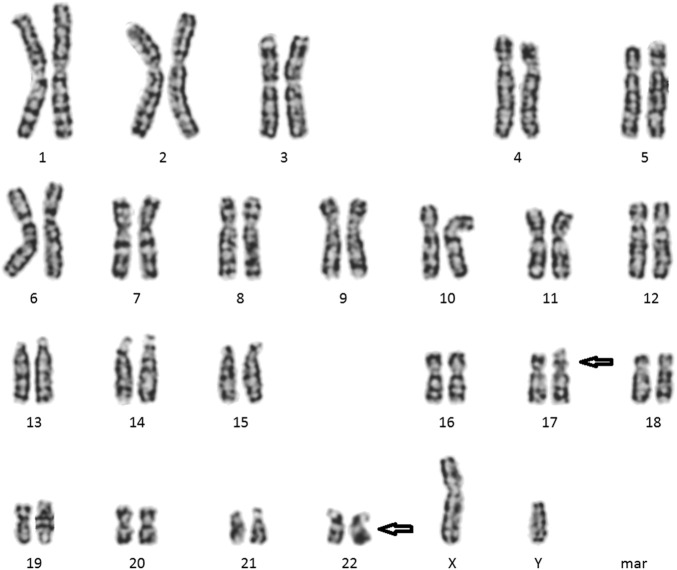
Cytogenetic images of chromosome karyotype (from peripheral blood sample): 46, XY, t (17; 22) (p11.2; q11.2).

**FIGURE 3:**
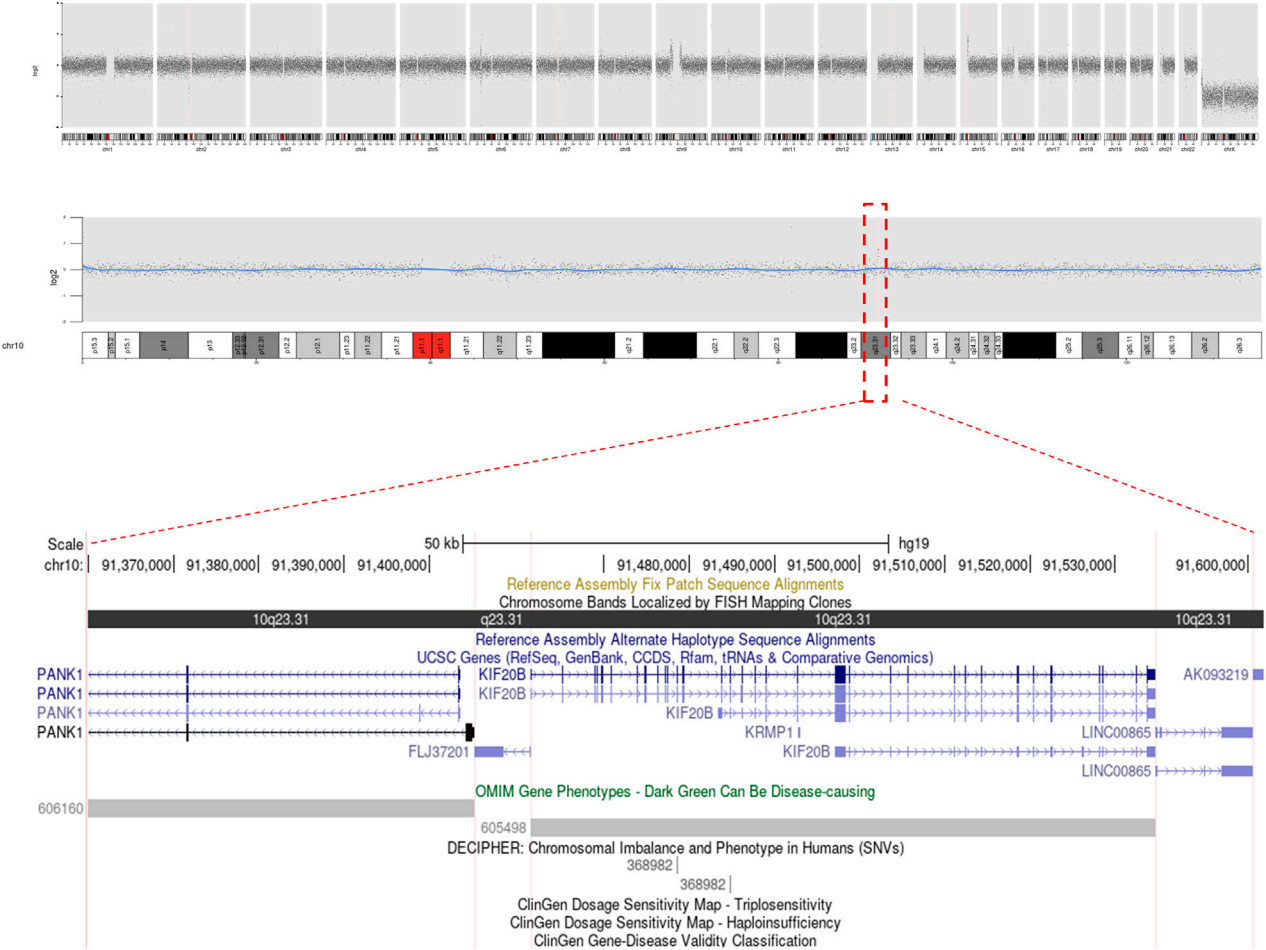
Copy number variation (CNV) analysis was perform on the case. A microduplication 10q23.31 (chr10:91371499-91596485) had been shown, affecting genes of FLJ37201, KIF20B, LINC00865, and PANK1.

Cytogenetic analyses (Giemsa banding) showed the karyotype of the patient to be 46, XY, t (17; 22) (p11.2; q11.2) (Figure 2) and that of his wife to be 46, XX. Microdeletion in azoospermia factor (AZF) regions (sy84, sy86, sy127, sy134, sy254, and sy255) was not found upon Y-chromosome analyses. CNVs showed a microduplication ∼224.98 kb in size, and the result described as seq [GRCh37] dup (10) (q23.31q23.31) chr10:g.91371499_91596485dup. However, this microduplication has not been mentioned in a public database previously (Figure 3). Unfortunately, the medical records from the patient’s parents were failed to be obtained. Hence, it is uncertain whether the two chromosomal rearrangements were *de novo* or inherited from his parents.

**FIGURE 4:**
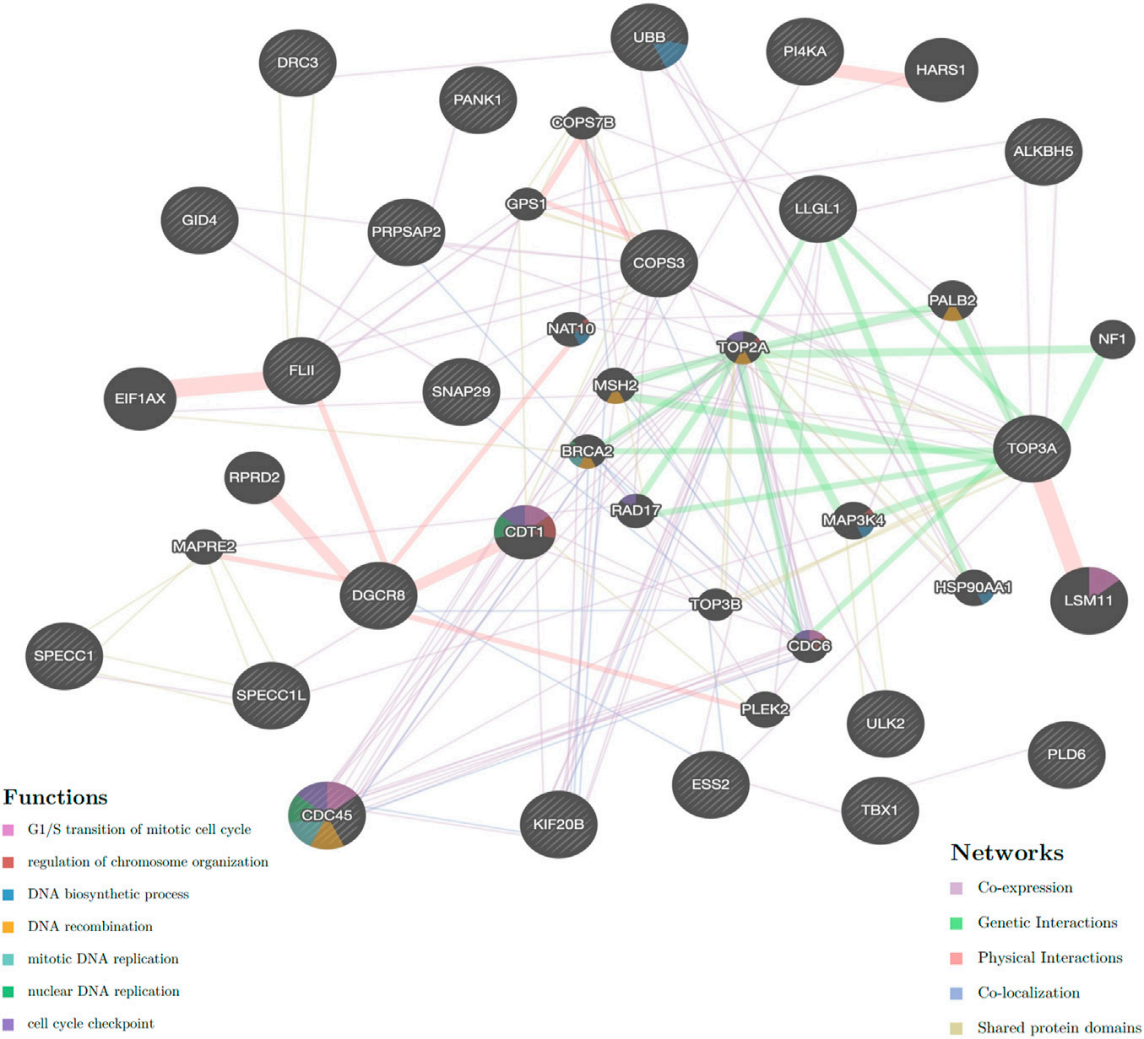
Network of genes located on the breakpoints of translocation and microduplication (available from GeneMANIA). Circles with a gray diagonal are the 21 genes in breakpoints; black circles are the 20 related genes; 3 genes (FLJ37201, LINC00865 and CCDC144NL-AS1) are found no interaction with these genes. Genetic linkages are drawn with different colors of lines, and the functions of these genes are present in color within circles. It has shown that genes of KIF20B, TOP3A, LLGL1 PRPSAP2, and CDC45 were closely associated with TOP2A expression, which has been found to be associated with the defective morphology of sperm heads.

In total, 24 genes located on the breakpoints of translocation and microduplication (Supplementary Table S3) were analyzed using GeneMANIA. The co-expression, genetic interactions, physical interactions, shared protein domains, and co-localization of these genes (with gray diagonal) are shown in Figure 4. Simultaneously, the functions of these genes were involved in cell cycle, regulation of chromosome organization, and the synthesis and replication of DNA. The cross-interaction of these genes reveals that they may contribute to spermatogenesis as a group.

## Discussion

### The Impact of Reciprocal Translocation on Genomic Instability and Spermatogenesis

Reciprocal translocation between chromosome 17 and chromosome 22 has been described in two male patients whose wives suffered repeated spontaneous abortions (Supplementary Table S2). Translocations involving chromosome 17 have been thought to be harmful for the fertility of the carrier (Geneix et al., 2002). Among the carriers of chromosome translocation, the prevalence of unbalanced gametes is high (about 81%) in the case of t (17; 22) (q11; q12) (Benet et al., 2005). This finding suggests that chromosome 17 and chromosome 22 might suffer abnormal meiotic segregation. The association between translocation, genes at the breakpoint, and reproductive pathogenic processes have not been specifically investigated.

Interestingly, chromosome-specific low-copy repeats on chromosomes 17p11.2 and 22q11.2 predispose these regions to recurrent rearrangement through non-allelic homologous recombination, which generates translocation, microduplication, and microdeletion events (Babcock et al., 2007) (Shaw et al., 2004) (Neira-Fresneda and Potocki, 2015) (Knoll et al., 1995) (Jawad et al., 2001). If translocation occurs, although the change in the genome may be balanced, it will also have a serious impact, such as t (15; 17) occurring in acute myeloid leukemia and t (9; 22) occurring in chronic myeloid leukemia (Kakizuka et al., 1991) (Tarkkanen et al., 1994).

Genome instability may play a role in male infertility. Recent epidemiological studies have identified an association between male infertility and cancer, and some “male infertility-associated cancer genes” have been established as risk factors in cancer progression (Nagirnaja et al., 2018). Some shared biological processes could explain the shared etiology of cancer and male infertility, such as cell survival, cell fate, and genome maintenance. Disruption in any of these pathways would be expected to lead to loss or damage of germ cells and the associated expression of male infertility. An interaction network was established according to the genes at the chromosome breakpoints in our patient (Figure 4). Interestingly, the genes for kinesin family member 20B (KIF20B), DNA topoisomerase III-alpha (TOP3A), LLGL scribble cell polarity complex component 1 (LLGL1), phosphoribosyl pyrophosphate synthetase-associated protein 2 (PRPSAP2), and cell division cycle 45 (CDC45) were closely associated with DNA topoisomerase II alpha (TOP2A) expression, which has been found to be associated with the defective morphology of sperm heads (Netherton et al., 2020). As found in our patient, the shape of the sperm head is the most common morphological abnormality reported.

The breakpoint of chromosome 17p11.2 (which is also the critical region of Smith–Magenis syndrome) contains genes involved in the pathogenesis of various cancers and processes of reproductive physiology. *TOP3A* is a protein-coding gene which catalyzes the transient breaking and rejoining of a single strand of DNA, thereby reducing the number of supercoils and altering the topology of DNA (Herbaux et al., 2012). *TOP3A* can modify the topologic states of genomic DNA and interact with the MLL gene to gain oncogenic characteristics in acute leukemia. Although it is linked primarily with bloom syndrome-like disorder and acute leukemia, *TOP3A* has been shown to participate in the meiotic cell cycle and meiotic recombination (Li and Wang, 1998). *LLGL1*, also called as Lgl1, encodes a protein that is similar to a tumor suppressor in *Drosophila* (Betschinger et al., 2003) (Zhu et al., 2020). It has a vital role in regulating proliferation, differentiation, and tissue organization in mammals and is involved in the regulation of establishment or maintenance of cell polarity (Jossin et al., 2017).

AlkB homolog 5 (ALKBH5) is an RNA demethylase highly expressed in the testes; it mediates m6A demethylation to regulate RNA metabolism and gene expression (Wang et al., 2020). *ALKBH5* plays a dual role in various cancers and reproductive system diseases and is described as a “cancer/testis gene.” *ALKBH5* loss results in impaired mouse fertility by affecting the metaphase stage of meiosis in the apoptosis of spermatocytes (Zheng et al., 2013). The effects of *ALKBH5* on sperm morphology have been demonstrated by controlling spermatogenic maturation, showing unmatured spermatids (round and elongated) in the stained sections of the testes and epididymis in *ALKBH5*-knockout mice (Tang et al., 2018). Dynein regulatory complex subunit 3 (DRC3) is a critical hub for controlling flagellar motility and is also a key component of the nexin–dynein regulatory complex (N-DRC). *DRC3* interacts with dynein g to regulate flagellar waveform, and mutations of *DRC3* cause a decrease in the movement speed of Chlamydomonas (Awata et al., 2015). In addition, COP9 signalosome subunit 3 (COPS3), is a component of the COP9 signalosome complex (CSN), which is involved in various cellular and developmental processes (Eunju Kim et al., 2011). Germ cells of CSN3-knockout mice cannot complete phase I of meiosis. Phospholipase D family member 6 (PLD6), also known as MitoPLD, is an endonuclease that plays a critical role in the biogenesis of PIWI-interacting RNA (piRNAs) during spermatogenesis (Watanabe et al., 2011). Destruction of the ubiquitin B (UBB) gene is related to low expression of many proteins involved in spermatogenesis and the reduction of germ cell numbers (Han et al., 2021). *UBB*-knockout mice are infertile because of structural destruction of the gonads and reproductive organs, which results in unformed gametes (Ryu et al., 2008). Some other genes, such as coiled coil domain containing 144NL antisense RNA 1(CCDC144NL-AS1), FLII actin remodeling protein (FLII), and unc-51 like autophagy activating kinase 2 (ULK2), are involved in important functions, such as cell proliferation (Wang et al., 2019), embryonic development (Jossin et al., 2017) (Yang et al., 2021), and autophagy (Chan et al., 2009). Genes exhibit multiple physiological and pathological functions depending on the tissue and/or cell type where they are expressed (Priyanka and Yenugu, 2021). Thus, genes that show high expression in testes might have roles in sperm function and male fertility. Genes for the GID complex subunit 4 homolog (GID4), sperm antigen with calponin homology and coiled coil domains 1(SPECC1), and *PRPSAP2* show high expression in testes. The changes of these genes located on the translocation breakpoint could lead to an increased risk of male infertility.

Genes mapped within or immediately adjacent to the breakpoint region of 22q11.2 are involved in hematological malignancies, such as chronic myeloid leukemia and other developmental disorders (Jawad et al., 2001) (Tarkkanen et al., 1994). Among them, the gene for sperm antigen with calponin homology and coiled coil domains 1 like (SPECC1L) shows the highest expression in testes compared with that in other tissues. *SPECC1L* was first identified to be disrupted by a balanced translocation t (1; 22) (21.3; q11.23) in a female patient with bilateral oromedial–canthal (Tessier IV) clefts (Dasouki et al., 1988). It encodes a cross-linking protein that interacts functionally with microtubules and the actin cytoskeleton, which is necessary for the adhesion and migration of cells (Saadi et al., 2011). Also, in cases of Opitz G/BBB syndrome, *SPECC1L* mutations can cause syndromic forms of facial clefting, which supports the original correlation to chromosome 22q11.2 (Kruszka et al., 2015). *CDC45* is a member of the pre-initiation complex in DNA replication, which is important in the early steps of DNA replication in eukaryotes. Biallelic mutations of *CDC45* cause a spectrum of phenotypes, including isolated short stature and craniosynostosis (Knapp et al., 2021) (Unolt et al., 2020), may promote hepatocellular carcinoma or non-small-cell lung cancer, and are correlated with a poor prognosis in patients (Lu et al., 2021) (Huang et al., 2019). Haploinsufficiency of the gene for T-box transcription factor-1 (TBX1) has been reported to cause abnormal growth and remodeling in the pharyngeal apparatus and other related structures (Lindsay et al., 2001), Similarly, *TBX1* disrupts orofacial and cranial nerve development by modifying retinoic acid-modulated anterior–posterior hindbrain differentiation (Yagi et al., 2003) (Verhagen et al., 2012). These disruptions likely contribute to dysphagia in infants and young children with DiGeorge syndrome.

Several other genes in the 22q11 region have been implicated in the pathogenesis of developmental disorders. The gene for phosphatidylinositol 4-kinase alpha (PI4KA) is implicated in the pathogenesis of cerebellar hypoplasia and arthrogryposis (Pagnamenta et al., 2015). Ess-2 splicing factor homolog (Ess2) (also termed Dgcr14) is a nuclear protein that bridges transcriptional regulators and spliceosomal complexes *via* distinct interacting domains, and *Ess2* might be involved in the pathogenesis of DiGeorge syndrome (Takada et al., 2018). The gene for synaptosome-associated protein 29 (SNAP29) has been shown to be involved in the formation of primary cilia, epidermal differentiation, membrane fusion, and autophagy. *SNAP29* has been implicated in recessive neurocutaneous cerebral dysgenesis, neuropathy, ichthyosis, and keratoderma (Martens et al., 2021). The gene for the DGCR8 microprocessor complex subunit (DGCR8) encodes a subunit of the microprocessor complex, which mediates the biogenesis of microRNAs from the primary microRNA transcript. *DGCR8* can enhance the migration and invasion of triple-negative breast cancer cells by targeting transforming growth factor-*β* (Cui et al., 2020). Hence, the genes mentioned previously appear to be associated with developmental disorders/retardation but might also affect spermatogenesis.

### The Microduplications’ Impact on Genomic Instability and Spermatogenesis

In addition to balanced reciprocal translocation, microarray analyses have revealed a novel 224.98-kb microduplication in chromosome 10q23.31. In total, four patients have been reported to have an overlap of microduplications in the genomic sequence of 10q23.31 (chr10:91371499-91596485) (Supplementary Table S4), and two of them have severe phenotypes (e.g., intellectual disability). Some other reports in chromosome 10 CNVs were indicated that impacted risk genes were responsible for different cellular processes, including cell signaling, sensing, and repair (Dai et al., 2013) (Li et al., 2018). Impairment of these genes is expected to disrupt the functions involved specifically with cellular development and may lead to diseases.

In the present case study, microduplication encompassed four genes: tigger transposable element derived 2 pseudogene (FLJ37201), *KIF20B*, long intergenic non-protein coding RNA 865 (LINC00865), and pantothenate kinase 1 (PANK1). None of these genes were identified in OMIM as causing diseases. However, whether any of these genes could be expression-sensitive and responsible for male infertility is not known. Pseudogenes were considered to be evolutionally conserved but have been found to act as “gene reservoirs” that might allow the genome to carry out novel functions effectively (Chen et al., 2020). It has been reported that more pseudogenes might be present in reproductive cells than in somatic cells (Noll et al., 2015). Moreover, transposable elements can create insults or rearrangements to chromosomes and affect gene expression, which can contribute to genomic innovations and genome instability (Klein and O'Neill, 2018). In this regard, with a high expression in testes, pseudogene *FLJ37201* could be a new candidate gene for recognizing the mechanisms of male infertility. *LINC00865* is a long noncoding RNA (lncRNA). lncRNAs have a sequence length of >200 nucleotides and are considered to be regulators of several cellular processes, particularly in tumorigenesis and cancer progression (Zhang et al., 2021). lncRNA expression can be regulated at gene and transcription levels and induce cell cycle arrest and apoptosis (Zhai et al., 2021). Unlike other lncRNAs, *LINC00865* has a high expression in testicular tissues and could be a cancer/testis-related gene.

*KIF20B* (previously called M-phase phosphoprotein 1) is a member of the kinesin-6 family. *KIF20B* is involved in the growth of the cerebral cortex and midbody organization of neural stem cells in mice (Janisch et al., 2018). *KIF20B* can accelerate or coordinate midbody maturation and regulate the late steps of maturation in a human cell line. *KIF20B* also has essential roles in multiple types of cancer, and could function as a cancer–testis antigen specific to bladder cancer in humans (Kanehira et al., 2007; Li et al., 2019). *KIF20B* also regulates cell proliferation, apoptosis, and tumor growth in hepatocellular carcinoma in association with the tumor protein p53 (TP53) (Liu et al., 2014). Similarly, *PANK1* is a transcriptional target for TP53, has a vital role in metabolic regulation, and modulates the energy balance in the adaptive immune system (Wang et al., 2013).

Evidence of a phenotypically well-defined syndrome resulting from 10q23.31 microduplication is lacking, and its clinical importance is unclear. In our case, the patient presented with teratozoospermia accompanied by asthenozoospermia. Even though no genetic information of the patient’s parents and aborted tissue was obtained and could not rule out other genetic (e.g., gene mutations) or non-genetic (e.g., environmental and multifactorial) factors, the karyotype of translocation and microduplication most likely represent a risk factor for the abnormal morphology of sperm and lack of motility under current conditions. The interaction network of 24 genes on breakpoints reveals that these genes may contribute to spermatogenesis as a group. Since the wife is naturally pregnant and her parameters of the physical examination are normal, the genetic defects of the patient are still the main cause of reproductive risk. Concerning future pregnancy, they are advised that there is an increased risk for karyotypically unbalanced fetuses and birth defects. And the pre-implantation genetic diagnosis (PGD) is recommended.

## Conclusion

A balanced reciprocal translocation t (17; 22) (p11.2; q11.2) and a microduplication in the region 10q23.31 were identified in our patient suffering from teratozoospermia. In total, 24 genes located on the breakpoints of translocation and microduplication were selected for their high expression in testicular tissues and associating with developmental disorders/retardation. The interaction of these genes might be risk factors for spermatogenesis, resulting in the phenotype of abnormal morphology of sperm and lack of motility. Combined with the balanced reciprocal translocation t (17; 22), 10q23.31 microduplication could have severe consequences for gametogenesis and could be transmitted across generations.

## Data Availability

The datasets for this article are not publicly available due to concerns regarding participant/patient anonymity. Requests to access the datasets should be directed to the corresponding author.

## References

[B1] Agarwal A. Baskaran S. Parekh N. Cho C.-L. Henkel R. Vij S. (2021). Male Infertility. The Lancet 397 (10271), 319–333. 10.1016/S0140-6736(20)32667-2 33308486

[B2] Awata J. Song K. Lin J. King S. M. Sanderson M. J. Nicastro D. (2015). DRC3 Connects the N-DRC to Dynein G to Regulate Flagellar Waveform. Mol. Biol. Cel 26 (15), 2788–2800. 10.1091/mbc.E15-01-0018 PMC4571338 26063732

[B3] Babcock M. Yatsenko S. Hopkins J. Brenton M. Cao Q. de Jong P. (2007). Hominoid Lineage Specific Amplification of Low-Copy Repeats on 22q11.2 (LCR22s) Associated with Velo-Cardio-Facial/digeorge Syndrome. Hum. Mol. Genet. 16 (21), 2560–2571. 10.1093/hmg/ddm197 17675367

[B4] Benet J. Oliver-Bonet M. Cifuentes P. Templado C. Navarro J. (2005). Segregation of Chromosomes in Sperm of Reciprocal Translocation Carriers: a Review. Cytogenet. Genome Res. 111 (3-4), 281–290. 10.1159/000086901 16192706

[B5] Betschinger J. Mechtler K. Knoblich J. A. (2003). The Par Complex Directs Asymmetric Cell Division by Phosphorylating the Cytoskeletal Protein Lgl. Nature 422 (6929), 326–330. 10.1038/nature01486 12629552

[B6] Chan E. Y. W. Longatti A. McKnight N. C. Tooze S. A. (2009). Kinase-inactivated ULK Proteins Inhibit Autophagy via Their Conserved C-Terminal Domains Using an Atg13-independent Mechanism. Mol. Cel Biol 29 (1), 157–171. 10.1128/MCB.01082-08 PMC2612494 18936157

[B7] Chen X. Wan L. Wang W. Xi W.-J. Yang A.-G. Wang T. (2020). Re-recognition of Pseudogenes: From Molecular to Clinical Applications. Theranostics 10 (4), 1479–1499. 10.7150/thno.40659 32042317 PMC6993246

[B8] Cui C. Y. Pan Q. W. Wang M. H. Ai X. Yan Y. Z. Tian Y. (2020). DGCR8 Promotes the Metastasis in Triple-Negative Breast Cancer by Epigenetically Regulating TGF-β. Eur. Rev. Med. Pharmacol. Sci. 24 (5), 2557–2563. 10.26355/eurrev_202003_20523 32196606

[B9] Dai L. Deng Y. Li N. Xie L. Mao M. Zhu J. (2013). Discontinuous Microduplications at Chromosome 10q24.31 Identified in a Chinese Family with Split Hand and Foot Malformation. BMC Med. Genet. 14, 45. 10.1186/1471-2350-14-45 23596994 PMC3637097

[B10] Dasouki M. Barr M. Jr. Erickson R. P. Cox B. (1988). Translocation (1;22) in a Child with Bilateral Oblique Facial Clefts. J. Med. Genet. 25 (6), 427–429. 10.1136/jmg.25.6.427 3398011 PMC1050514

[B11] Eunju Kim E. Yoon S.-J. Kim E.-Y. Kim Y. Lee H.-S. Kim K.-H. (2011). Function of COP9 Signalosome in Regulation of Mouse Oocytes Meiosis by Regulating MPF Activity and Securing Degradation. PLoS One 6 (10), e25870. 10.1371/journal.pone.0025870 21991377 PMC3185060

[B12] Franz M. Rodriguez H. Lopes C. Zuberi K. Montojo J. Bader G. D. (2018). GeneMANIA Update 2018. Nucleic Acids Res. 46 (W1), W60–W64. 10.1093/nar/gky311 29912392 PMC6030815

[B13] Geneix A. Schubert B. Force A. Rodet K. Briancon G. Boucher D. (2002). Sperm Analysis by FISH in a Case of T(17; 22) (Q11; Q12) Balanced Translocation: Case Report. Hum. Reprod. 17 (2), 325–331. 10.1093/humrep/17.2.325 11821272

[B14] Han B. Jung B.-K. Park S.-H. Song K. J. Anwar M. A. Ryu K.-Y. (2021). Polyubiquitin Gene Ubb Is Required for Upregulation of Piwi Protein Level during Mouse Testis Development. Cell Death Discov. 7 (1), 194. 10.1038/s41420-021-00581-2 34312369 PMC8313548

[B15] Herbaux C. Poulain S. Meyer C. Marschalek R. Renneville A. Fernandes J. (2012). TOP3A, a New Partner Gene Fused to MLL in an Adult Patient with De Novo Acute Myeloid Leukaemia. Br. J. Haematol. 157 (1), 128–131. 10.1111/j.1365-2141.2011.08908.x 22050635

[B16] Hofherr S. E. Wiktor A. E. Kipp B. R. Dawson D. B. Van Dyke D. L. (2011). Clinical Diagnostic Testing for the Cytogenetic and Molecular Causes of Male Infertility: the Mayo Clinic Experience. J. Assist. Reprod. Genet. 28 (11), 1091–1098. 10.1007/s10815-011-9633-6 21912980 PMC3224174

[B17] Huang J. Li Y. Lu Z. Che Y. Sun S. Mao S. (2019). Analysis of Functional Hub Genes Identifies CDC45 as an Oncogene in Non-small Cell Lung Cancer - a Short Report. Cell Oncol. 42 (4), 571–578. 10.1007/s13402-019-00438-y PMC12994300 30887286

[B18] Janisch K. M. McNeely K. C. Dardick J. M. Lim S. H. Dwyer N. D. (2018). Kinesin-6 KIF20B Is Required for Efficient Cytokinetic Furrowing and Timely Abscission in Human Cells. Mol. Biol. Cel 29 (2), 166–179. 10.1091/mbc.E17-08-0495 PMC5909929 29167382

[B19] Jawad A. F. McDonald-Mcginn D. M. Zackai E. Sullivan K. E. (2001). Immunologic Features of Chromosome 22q11.2 Deletion Syndrome (DiGeorge Syndrome/velocardiofacial Syndrome). J. Pediatr. 139 (5), 715–723. 10.1067/mpd.2001.118534 11713452

[B20] Ji Won Kim J. W. Chang E. M. Song S.-H. Park S. H. Yoon T. K. Shim S. H. (2011). Complex Chromosomal Rearrangements in Infertile Males: Complexity of Rearrangement Affects Spermatogenesis. Fertil. Sterility 95 (1), 349–352. 10.1016/j.fertnstert.2010.08.014 20864097

[B21] Jossin Y. Lee M. Klezovitch O. Kon E. Cossard A. Lien W.-H. (2017). Llgl1 Connects Cell Polarity with Cell-Cell Adhesion in Embryonic Neural Stem Cells. Dev. Cel 41 (5), 481–495. 10.1016/j.devcel.2017.05.002 PMC5519327 28552558

[B22] Kakizuka A. Miller W. H. Jr. Umesono K. Warrell R. P. Jr. Frankel S. R. Murty V. V. V. S. (1991). Chromosomal Translocation T(15;17) in Human Acute Promyelocytic Leukemia Fuses RARα with a Novel Putative Transcription Factor, PML. Cell 66 (4), 663–674. 10.1016/0092-8674(91)90112-c 1652368

[B23] Kanehira M. Katagiri T. Shimo A. Takata R. Shuin T. Miki T. (2007). Oncogenic Role of MPHOSPH1, a Cancer-Testis Antigen Specific to Human Bladder Cancer. Cancer Res. 67 (7), 3276–3285. 10.1158/0008-5472.CAN-06-3748 17409436

[B24] Klein S. J. O’Neill R. J. (2018). Transposable Elements: Genome Innovation, Chromosome Diversity, and Centromere Conflict. Chromosome Res. 26 (1-2), 5–23. 10.1007/s10577-017-9569-5 29332159 PMC5857280

[B25] Knapp K. M. Fellows B. Aggarwal S. Dalal A. Bicknell L. S. (2021). A Synonymous Variant in a Non-canonical Exon of CDC45 Disrupts Splicing in Two Affected Sibs with Meier-Gorlin Syndrome with Craniosynostosis. Eur. J. Med. Genet. 64 (4), 104182. 10.1016/j.ejmg.2021.104182 33639314

[B26] Knoll J. H. M. Asamoah A. Pletcher B. A. Wagstaff J. (1995). Interstitial Duplication of Proximal 22q: Phenotypic Overlap with Cat Eye Syndrome. Am. J. Med. Genet. 55 (2), 221–224. 10.1002/ajmg.1320550214 7717422

[B27] Krausz C. Riera-Escamilla A. (2018). Genetics of Male Infertility. Nat. Rev. Urol. 15 (6), 369–384. 10.1038/s41585-018-0003-3 29622783

[B28] Kruszka P. Li D. Harr M. H. Wilson N. R. Swarr D. McCormick E. M. (2015). Mutations inSPECC1L, Encoding Sperm Antigen with Calponin Homology and Coiled-Coil Domains 1-like, Are Found in Some Cases of Autosomal Dominant Opitz G/BBB Syndrome. J. Med. Genet. 52 (2), 104–110. 10.1136/jmedgenet-2014-102677 25412741 PMC4393015

[B29] Li W. Wang J. C. (1998). Mammalian DNA Topoisomerase IIIα Is Essential in Early Embryogenesis. Proc. Natl. Acad. Sci. U.S.A. 95 (3), 1010–1013. 10.1073/pnas.95.3.1010 9448276 PMC18654

[B30] Li J.-J. Chen Y.-q. Fan L.-L. Jin J.-Y. Guo S. Xiang R. (2018). Microduplication of 10q26.3 in a Chinese Hypertriglyceridemia Patient. Mol. Cell Probes 37, 28–31. 10.1016/j.mcp.2017.11.002 29129660

[B31] Li Z. Y. Wang Z. X. Li C. C. (2019). Kinesin Family Member 20B Regulates Tongue Cancer Progression by Promoting Cell Proliferation. Mol. Med. Rep. 19 (3), 2202–2210. 10.3892/mmr.2019.9851 30664160 PMC6390006

[B32] Lindsay E. A. Vitelli F. Su H. Morishima M. Huynh T. Pramparo T. (2001). Tbx1 Haploinsufficiency in the DiGeorge Syndrome Region Causes Aortic Arch Defects in Mice. Nature 410 (6824), 97–101. 10.1038/35065105 11242049

[B65] Liu X. Zhou Y. Liu X. Peng A. Gong H. Huang L. (2014). MPHOSPH1: A Potential Therapeutic Target for Hepatocellular Carcinoma. Cancer Res. 74 (22), 6623–6634. 10.1158/0008-5472.CAN-14-1279 25269478

[B33] Lu H.-P. Du X.-F. Li J.-D. Huang S.-N. He R.-Q. Wu H.-Y. (2021). Expression of Cell Division Cycle Protein 45 in Tissue Microarrays and the CDC45 Gene by Bioinformatics Analysis in Human Hepatocellular Carcinoma and Patient Outcomes. Med. Sci. Monit. 27, e928800. 10.12659/MSM.928800 33622998 PMC7919231

[B34] Manvelyan M. Schreyer I. Höls-Herpertz I. Köhler S. Niemann R. Hehr U. (2007). Forty-eight New Cases with Infertility Due to Balanced Chromosomal Rearrangements: Detailed Molecular Cytogenetic Analysis of the 90 Involved Breakpoints. Int. J. Mol. Med. 19 (6), 855–864. 10.3892/ijmm.19.6.855 17487417

[B35] Martens M. C. Edelkamp J. Seebode C. Schäfer M. Stählke S. Krohn S. (2021). Generation and Characterization of a CRISPR/Cas9-Mediated SNAP29 Knockout in Human Fibroblasts. Int. J. Mol. Sci. 22 (10), 5293. 10.3390/ijms22105293 34069872 PMC8157373

[B36] Nagirnaja L. Aston K. I. Conrad D. F. (2018). Genetic Intersection of Male Infertility and Cancer. Fertil. Sterility 109 (1), 20–26. 10.1016/j.fertnstert.2017.10.028 PMC5761685 29307395

[B37] Neira-Fresneda J. Potocki L. (2015). Neurodevelopmental Disorders Associated with Abnormal Gene Dosage: Smith-Magenis and Potocki-Lupski Syndromes. J. Pediatr. Genet. 04 (3), 159–167. 10.1055/s-0035-1564443 PMC4918721 27617127

[B38] Netherton J. Ogle R. A. Hetherington L. Silva Balbin Villaverde A. I. Hondermarck H. Baker M. A. (2020). Proteomic Analysis Reveals that Topoisomerase 2A Is Associated with Defective Sperm Head Morphology. Mol. Cell Proteomics 19 (3), 444–455. 10.1074/mcp.RA119.001626 31848259 PMC7050105

[B39] Noll A. Raabe C. A. Churakov G. Brosius J. Schmitz J. (2015). Ancient Traces of Tailless Retropseudogenes in Therian Genomes. Genome Biol. Evol. 7 (3), 889–900. 10.1093/gbe/evv040 25724209 PMC5322556

[B40] Organization W. H. (2010). WHO Laboratory Manual for the Examination and Processing of Human Semen. 5th ed. Geneva, Switzerland: World Health Organization.

[B41] Pagnamenta A. T. Howard M. F. Wisniewski E. Popitsch N. Knight S. J. L. Keays D. A. (2015). Germline Recessive Mutations in PI4KA Are Associated with Perisylvian Polymicrogyria, Cerebellar Hypoplasia and Arthrogryposis. Hum. Mol. Genet. 24 (13), 3732–3741. 10.1093/hmg/ddv117 25855803 PMC4459391

[B42] Priyanka P. P. Yenugu S. (2021). Coiled-Coil Domain-Containing (CCDC) Proteins: Functional Roles in General and Male Reproductive Physiology. Reprod. Sci. 28, 2725–2734. 10.1007/s43032-021-00595-2 33942254

[B43] Jean McGowan-Jordan R. J. H. Moore S. (Editors) (2020). ISCN 2020: An International System for Human Cytogenomic Nomenclature. 2020 ed (Basel, Switzerland: S. Karger AG).

[B44] Ryu K.-Y. Sinnar S. A. Reinholdt L. G. Vaccari S. Hall S. Garcia M. A. (2008). The Mouse Polyubiquitin Gene Ubb Is Essential for Meiotic Progression. Mol. Cel Biol 28 (3), 1136–1146. 10.1128/MCB.01566-07 PMC2223379 18070917

[B45] Saadi I. Alkuraya F. S. Gisselbrecht S. S. Goessling W. Cavallesco R. Turbe-Doan A. (2011). Deficiency of the Cytoskeletal Protein SPECC1L Leads to Oblique Facial Clefting. Am. J. Hum. Genet. 89 (1), 44–55. 10.1016/j.ajhg.2011.05.023 21703590 PMC3135813

[B46] Shaw C. J. Withers M. A. Lupski J. R. (2004). Uncommon Deletions of the Smith-Magenis Syndrome Region Can Be Recurrent when Alternate Low-Copy Repeats Act as Homologous Recombination Substrates. Am. J. Hum. Genet. 75 (1), 75–81. 10.1086/422016 15148657 PMC1182010

[B47] Simoni M. Bakker E. Eurlings M. C. Matthijs G. Moro E. Muller C. R. (1999). Laboratory Guidelines for Molecular Diagnosis of Y-Chromosomal Microdeletions. Int. J. Androl. 22 (5), 292–299. 10.1046/j.1365-2605.1999.00193.x 10509229

[B48] Takada I. Tsuchiya M. Yanaka K. Hidano S. Takahashi S. Kobayashi T. (2018). Ess2 Bridges Transcriptional Regulators and Spliceosomal Complexes via Distinct Interacting Domains. Biochem. Biophysical Res. Commun. 497 (2), 597–604. 10.1016/j.bbrc.2018.02.110 29454968

[B49] Tang C. Klukovich R. Peng H. Wang Z. Yu T. Zhang Y. (2018). ALKBH5-dependent m6A Demethylation Controls Splicing and Stability of Long 3′-UTR mRNAs in Male Germ Cells. Proc. Natl. Acad. Sci. U.S.A. 115 (2), E325–E333. 10.1073/pnas.1717794115 29279410 PMC5777073

[B50] Tarkkanen M. Knuutila S. Wiklund T. Virolainen M. Elomaa I. (1994). Dedifferentiated Chondrosarcoma with T(9;22)(q34;q 11-12). Genes Chromosom. Cancer 9 (2), 136–140. 10.1002/gcc.2870090210 7513544

[B51] Uhlén M. Fagerberg L. Hallström B. M. Lindskog C. Oksvold P. Mardinoglu A. (2015). Proteomics. Tissue-Based Map of the Human Proteome. Science 347 (6220), 1260419. 10.1126/science.1260419 25613900

[B52] Unolt M. Kammoun M. Nowakowska B. Graham G. E. Crowley T. B. Hestand M. S. (2020). Pathogenic Variants in CDC45 on the Remaining Allele in Patients with a Chromosome 22q11.2 Deletion Result in a Novel Autosomal Recessive Condition. Genet. Med. 22 (2), 326–335. 10.1038/s41436-019-0645-4 31474763 PMC7197230

[B53] Verhagen J. M. A. Diderich K. E. M. Oudesluijs G. Mancini G. M. S. Eggink A. J. Verkleij-Hagoort A. C. (2012). Phenotypic Variability of Atypical 22q11.2 Deletions Not includingTBX1. Am. J. Med. Genet. 158A (10), 2412–2420. 10.1002/ajmg.a.35517 22893440

[B54] Wang S.-J. Yu G. Jiang L. Li T. Lin Q. Tang Y. (2013). p53-Dependent Regulation of Metabolic Function through Transcriptional Activation of Pantothenate Kinase-1 Gene. Cell Cycle 12 (5), 753–761. 10.4161/cc.23597 23343762 PMC3610723

[B55] Wang Y. Guo B. Xiao Z. Lin H. Zhang X. Song Y. (2019). Long Noncoding RNA CCDC144NL-AS1 Knockdown Induces Naïve-like State Conversion of Human Pluripotent Stem Cells. Stem Cel Res Ther 10 (1), 220. 10.1186/s13287-019-1323-9 PMC6664583 31358062

[B56] Wang J. Wang J. Gu Q. Ma Y. Yang Y. Zhu J. (2020). The Biological Function of m6A Demethylase ALKBH5 and its Role in Human Disease. Cancer Cel Int 20, 347. 10.1186/s12935-020-01450-1 PMC7388453 32742194

[B57] Watanabe T. Chuma S. Yamamoto Y. Kuramochi-Miyagawa S. Totoki Y. Toyoda A. (2011). MITOPLD Is a Mitochondrial Protein Essential for Nuage Formation and piRNA Biogenesis in the Mouse Germline. Dev. Cel 20 (3), 364–375. 10.1016/j.devcel.2011.01.005 PMC3062204 21397847

[B58] Yagi H. Furutani Y. Hamada H. Sasaki T. Asakawa S. Minoshima S. (2003). Role of TBX1 in human del22q11.2 syndrome. The Lancet 362 (9393), 1366–1373. 10.1016/s0140-6736(03)14632-6 14585638

[B59] Yang G. N. Ahangar P. Strudwick X. L. Kopecki Z. Cowin A. J. (2021). Overexpression of Flii during Murine Embryonic Development Increases Symmetrical Division of Epidermal Progenitor Cells. Int. J. Mol. Sci. 22 (15), 8235. 10.3390/ijms22158235 34361001 PMC8348627

[B60] Zahn-Zabal M. Michel P.-A. Gateau A. Nikitin F. Schaeffer M. Audot E. (2020). The neXtProt Knowledgebase in 2020: Data, Tools and Usability Improvements. Nucleic Acids Res. 48 (D1), D328–D334. 10.1093/nar/gkz995 31724716 PMC7145669

[B61] Zhai S. Xu Z. Xie J. Zhang J. Wang X. Peng C. (2021). Epigenetic Silencing of LncRNA LINC00261 Promotes C-Myc-Mediated Aerobic Glycolysis by Regulating miR-222-3p/HIPK2/ERK axis and Sequestering IGF2BP1. Oncogene 40 (2), 277–291. 10.1038/s41388-020-01525-3 33122827 PMC7808938

[B62] Zhang M. Gao F. Yu X. Zhang Q. Sun Z. He Y. (2021). LINC00261: a Burgeoning Long Noncoding RNA Related to Cancer. Cancer Cel Int 21 (1), 274. 10.1186/s12935-021-01988-8 PMC8141177 34022894

[B63] Zheng G. Dahl J. A. Niu Y. Fedorcsak P. Huang C.-M. Li C. J. (2013). ALKBH5 Is a Mammalian RNA Demethylase that Impacts RNA Metabolism and Mouse Fertility. Mol. Cel 49 (1), 18–29. 10.1016/j.molcel.2012.10.015 PMC3646334 23177736

[B64] Zhu Y.-X. Li C. H. Li G. Feng H. Xia T. Wong C. H. (2020). LLGL1 Regulates Gemcitabine Resistance by Modulating the ERK-SP1-OSMR Pathway in Pancreatic Ductal Adenocarcinoma. Cell Mol. Gastroenterol. Hepatol. 10 (4), 811–828. 10.1016/j.jcmgh.2020.06.009 32615164 PMC7505810

