# Supplementary material for: Case Report: Balanced Reciprocal Translocation t (17; 22) (p11.2; q11.2) and 10q23.31 Microduplication in an Infertile Male Patient Suffering From Teratozoospermia
Source: Front Genet. 2022 May 26;13:797813. doi: 10.3389/fgene.2022.797813 (PMC9204271; doi:10.3389/fgene.2022.797813)
Supplement: Supplementary file 1 [file Table1.DOCX]

**Supplementary Table 1. The clinical and phenotypic properties of the patient**

| Parameter | Value (normal range) |
| --- | --- |
| Height | 169cm |
| Body weight | 66kg |
| Body Mass Index(BMI) | 23.1 |
| Smoking | no |
| Alcoholism | no |
| Testicular volume | ~18ml |
| Lateral spermatic vein | normal |
| Follicle-stimulating hormone(FSH) | 3.53mIU/mL (1.50–12.4) |
| Luteinizing hormone(LH) | 3.70mIU/mL (1.7–8.6) |
| Testosterone(TESTO) | 4.42ng/mL (2.49–8.36) |
| Estradiol(E2) | 26.25pg/mL (25.8–60.7) |
| Prolactin(PROL) | 12.51ng/mL (4.04–15.2) |
| Y-chromosome microdeletion | Not detected  (sy84, sy86, sy127, sy134, sy254, sy255) |
| Sperm concentration(million/ml) | 20.10million/ml (≥15) |
| Total sperm motility (%) | 35.84% (≥40%) |
| Progressive motility (%) | 27.7% (≥32%) |
| Abnormal morphology rate (%) | 100% (<96%) |
