# Supplementary material for: Case Report: Balanced Reciprocal Translocation t (17; 22) (p11.2; q11.2) and 10q23.31 Microduplication in an Infertile Male Patient Suffering From Teratozoospermia
Source: Front Genet. 2022 May 26;13:797813. doi: 10.3389/fgene.2022.797813 (PMC9204271; doi:10.3389/fgene.2022.797813)
Supplement: Supplementary file 2 [file Table2.DOCX]

**Supplementary Table 2.** **Summary of reported reciprocal translocation t(17;22)**

| **Karyotype of the carrier** | **Clinical features of the translocation** | **Reference** |
| --- | --- | --- |
| 46,XY,inv(1)(p13:q21),t(17;22)(p11:p11) | Recurrent abortion | ([Kim et al. 2011](#_ENREF_26)) |
| 46,XY,t(17;22)(q11:q12) | Miscarriage;  Oligoteratozoospermia | ([Geneix et al. 2002](#_ENREF_18)) |
