# Supplementary material for: Case Report: Balanced Reciprocal Translocation t (17; 22) (p11.2; q11.2) and 10q23.31 Microduplication in an Infertile Male Patient Suffering From Teratozoospermia
Source: Front Genet. 2022 May 26;13:797813. doi: 10.3389/fgene.2022.797813 (PMC9204271; doi:10.3389/fgene.2022.797813)
Supplement: Supplementary file 3 [file Table3.docx]

**Supplementary Table 3. Genes located at the breakpoint(17p11.2 and 22q11.2) and the duplicated region(10q23.31)**

| **Number** | **Genes** | **Description** | **Location** | **Expression (the first / the second)** | **function** |
| --- | --- | --- | --- | --- | --- |
| 1 | TOP3A | DNA topoisomerase III alpha | 17p11.2 | Testis / Bone marrow | Chromosome separation (GO:0051304);  Meiotic cell cycle (GO:0051321);  DNA topoisomerase activity (GO:0003916) |
| 2 | LLGL1 | LLGL scribble cell polarity complex component 1 | 17p11.2 | Brain / Testis | Regulation of establishment or maintenance of cell polarity (GO:0032878);  Regulation of protein secretion (GO:0050708) |
| 3 | ALKBH5 | alkB homolog 5 | 17p11.2 | Testis / Brain | Spermatogenesis(GO:0007283);  Cell differentiation(GO:0030154) |
| 4 | DRC3 | dynein regulatory complex subunit 3 | 17p11.2 | Testis / Thyroid | Protein binding (GO:0005515) |
| 5 | COPS3 | COP9 signalosome subunit 3 | 17p11.2 | Testis / Bone marrow | In utero embryonic development (GO:0001701);  Protein binding (GO:0005515);  Protein deneddylation (GO:0000338) |
| 6 | PLD6 | phospholipase D family member 6 | 17p11.2 | Testis / Prostate | DNA methylation involved in gamete generation (GO:0043046);  Meiotic cell cycle (GO:0051321);  Spermatid development (GO:0007286) |
| 7 | UBB | ubiquitin B | 17p11.2 | Liver / Testis | Male meiosis I (GO:0007141);  Seminiferous tubule development (GO:0072520);  Positive regulation of intrinsic apoptotic signaling pathway by p53 class mediator (GO:1902255) |
| 8 | CCDC144NL-AS1 | coiled-coil domain containing 144NL antisense RNA 1 | 17p11.2 | Testis / Placenta | Knockdown of CCDC144NL-AS1 was related to conversion of human pluripotent stem cells; Knockdown of CCDC144NL-AS1 dramatically altered the distribution of cytoskeletal filamentous actin (F-actin) stress fibers compared to the negative control group treatment. (Wang et al., 2019) |
| 9 | FLII | FLII actin remodeling protein | 17p11.2 | Testis / Spleen | Actin binding (GO:0003779);  Enables protein binding (GO:0005515) |
| 10 | ULK2 | unc-51 like autophagy activating kinase 2 | 17p11.2 | Testis / Brain | Protein serine/threonine kinase activity (GO:0004674);  Cellular response to DNA damage stimulus (GO:0006974);  Neuron projection development (GO:0031175);  Autophagy (GO:0006914) |
| 11 | GID4 | GID complex subunit 4 homolog | 17p11.2 | Testis / Esophagus | Ubiquitin protein ligase activity (GO:0061630);  Protein ubiquitination (GO:0016567);  proteasome-mediated ubiquitin-dependent protein catabolic process (GO:0043161) |
| 12 | SPECC1 | sperm antigen with calponin homology and coiled-coil domains 1 | 17p11.2 | Brain / Testis | Actin cytoskeleton organization (GO:0030036 ) |
| 13 | PRPSAP2 | phosphoribosyl pyrophosphate synthetase associated protein 2 | 17p11.2 | Lymph node / Testis | Protein binding (GO:0005515);  Negative regulation of catalytic activity (GO:0043086);  5-phosphoribose 1-diphosphate biosynthetic process (GO:0006015) |
| 14 | SPECC1L | sperm antigen with calponin homology and coiled-coil domains 1 like | 22q11.2 | Testis / Thyroid | Protein binding (GO:0005515);  Cell adhesion (GO:0007155);  Cell cycle (GO:0007049);  Cell division (GO:0051301) |
| 15 | CDC45 | cell division cycle 45 | 22q11.2 | Testis / Bone marrow | Single-stranded DNA binding (GO:0003697);  Mitotic DNA replication preinitiation complex assembly (GO:1902977);  Double-strand break repair via break-induced replication (GO:0000727);  DNA replication checkpoint signaling (GO:0000076) |
| 16 | TBX1 | T-box transcription factor 1 | 22q11.2 | Testis / Prostate | DNA-binding transcription factor activity, RNA polymerase II-specific (GO:0000981);  Cell population proliferation (GO:0008283);  Negative regulation of cell differentiation (GO:0045596);  Positive regulation of transcription, DNA-templated (GO:0045893) |
| 17 | PI4KA | phosphatidylinositol 4-kinase alpha | 22q11.2 | Brain / Testis | Phosphatidylinositol kinase activity (GO:0052742);  Signal transduction (GO:0007165);  Phosphatidylinositol-mediated signaling (GO:0048015) |
| 18 | Ess2 | ess-2 splicing factor homolog | 22q11.2 | Testis / Bone marrow | Protein binding (GO:0005515);  mRNA splicing, via spliceosome (GO:0000398);  Nervous system development (GO:0007399) |
| 19 | SNAP29 | synaptosome associated protein 29 | 22q11.2 | Testis / Brain | Protein binding (GO:0005515);  Protein transport (GO:0015031);  Synaptic vesicle fusion to presynaptic active zone membrane (GO:0031629) |
| 20 | DGCR8 | DGCR8 microprocessor complex subunit | 22q11.2 | Testis / Placenta | Primary miRNA processing (GO:0031053);  Protein homodimerization activity (GO:0042803);  Protein-RNA adaptor activity (GO:0140517) |
| 21 | FLJ37201 | tigger transposable element derived 2 pseudogene | 10q23.31 | Testis / Thyroid | More present in reproductive cells (Noll et al., 2015);  Involving in genomic innovations and genome instability (Klein and O'Neill, 2018). |
| 22 | LINC00865 | long intergenic non-protein coding RNA 865 | 10q23.31 | Testis / Urinary bladder | Regulator of cellular processes (Zhang et al., 2021). |
| 23 | KIF20B | kinesin family member 20B | 10q23.31 | Testis / Lymph node | Cell division (GO:0051301);  Positive regulation of cell population proliferation (GO:0008284);  Positive regulation of cytokinesis (GO:0032467);  Involved in cell cycle (GO:0007049);  Involved in regulation of mitotic nuclear division (GO:0007088);  Involved in positive regulation of mitotic cytokinetic process (GO:1903438);  Involved in regulation of cell cycle (GO:0051726);  Involved in regulation of establishment of cell polarity (GO:2000114) |
| 24 | PANK1 | pantothenate kinase 1 | 10q23.31 | Liver / Kidney | Coenzyme A biosynthetic process (GO:0015937);  Phosphorylation (GO:0016310);  Protein homodimerization activity (GO:0042803);  Pantothenate kinase activity (GO:0004594) |
