# Supplementary material for: Case Report: Balanced Reciprocal Translocation t (17; 22) (p11.2; q11.2) and 10q23.31 Microduplication in an Infertile Male Patient Suffering From Teratozoospermia
Source: Front Genet. 2022 May 26;13:797813. doi: 10.3389/fgene.2022.797813 (PMC9204271; doi:10.3389/fgene.2022.797813)
Supplement: Supplementary file 4 [file Table4.DOCX]

**Supplementary Table 4.** **The reported cases with duplication overlap in genomic sequence of 10q23.31(chr10:91371499-91596485)**

| DECIPHER Patient | Sex | Location | Type | Size | Inheritance/ Genotype | Pathogenicity /Contribution | Phenotype(s) |
| --- | --- | --- | --- | --- | --- | --- | --- |
| 252137 | 46,XY | chr10:91344168-93973279 | Duplication | 2.63Mb | Inherited from normal parent  Heterozygous | Uncertain | Delayed puberty, Obesity,  High palate, Upslanted palpebral fissure,  Abnormal foot morphology  Autism, Cognitive impairment,  Intellectual disability |
| 341717 | 46,XY | chr10:91860336-100377991 | Duplication | 8.52Mb | Unknown  Heterozygous | Pathogenic | Emotional lability, Growth delay, Intellectual disability |
| 273411 | 46,XX | chr10:91127807-91626682 | Duplication | 498.88kb | Unknown  Heterozygous | Uncertain | Unknown |
| 433265 | 46,XX | chr10:88708340-94958183 | Duplication | 6.25Mb | Unknown  Heterozygous | Likely pathogenic | Unknown |
